# Livestock-associated methicillin-resistant *Staphylococcus aureus* epidemiology, genetic diversity, and clinical characteristics in an urban region

**DOI:** 10.3389/fmicb.2022.875775

**Published:** 2022-12-14

**Authors:** Maria M. Konstantinovski, Leo M. Schouls, Sandra Witteveen, Eric C. J. Claas, Margriet E. Kraakman, Jayant Kalpoe, Eva Mattson, David J. Hetem, Erika P. M. van Elzakker, Jos Kerremans, Vishal Hira, Thijs Bosch, Jairo Gooskens

**Affiliations:** ^1^Department of Medical Microbiology, Leiden University Medical Center, Leiden, Netherlands; ^2^Department of Microbiology, Medical Laboratories, Reinier de Graaf Groep, Delft, Netherlands; ^3^Center for Infectious Diseases Research, Diagnostics and Laboratory Surveillance, National Institute for Public Health and the Environment (RIVM), Bilthoven, Netherlands; ^4^Department of Medical Microbiology, Regional Laboratory of Public Health Kennemerland, Haarlem, Netherlands; ^5^Department of Medical Microbiology, Haaglanden Medical Center, The Hague, Netherlands; ^6^Laboratory of Medical Microbiology, HagaZiekenhuis, The Hague, Netherlands; ^7^Department of Medical Microbiology, Alrijne Hospital, Leiderdorp, Netherlands; ^8^Department of Medical Microbiology and Infection Prevention, Groene Hart Ziekenhuis, Gouda, Netherlands

**Keywords:** cgMLST clustering, one-health, antimicrobial surveillance, LA-MRSA CC398, whole-genome sequencing, Staphylococcal infection/epidemiology, MRSA, *Staphylococcus aureus*

## Abstract

**Objectives:**

While Livestock-associated methicillin-resistant *Staphylococcus aureus* (LA-MRSA), defined as CC398, is a well-known pathogen among those working with livestock, there are indications that LA-MRSA prevalence among the general population is increasing. However, the clinical impact in urban areas remains unknown. The aim of this study was to assess the genetic epidemiology and clinical characteristics of LA-MRSA in an urban area with a limited livestock population.

**Methods:**

In this retrospective study, we evaluated LA-MRSA strains that were collected between 2014 and 2018 from patients who received clinical care in a single urban area in Netherlands. Patient files were assessed for livestock exposure data, clinical findings, and contact tracing information. Next-generation sequencing (NGS) analysis in combination with wgMLST was conducted to assess genetic diversity and relatedness and to detect virulence and resistance genes.

**Results:**

LA-MRSA strains were cultured from 81 patients, comprising 12% of all the MRSA strains found in seven study laboratories between 2014 and 2018. No livestock link was found in 76% of patients (*n* = 61), and 28% of patients (*n* = 23) had an infection, mostly of the skin or soft tissue. Contact tracing had been initiated in 14 cases, leading to the identification of two hospital transmissions: a cluster of 9 cases and one of 2 cases. NGS data were available for 91% (*n* = 75) of the patients. wgMLST confirmed the clusters detected *via* contact tracing (*n* = 2) and identified 5 additional clusters without a known epidemiological link. Relevant resistance and virulence findings included the PVL virulence gene (3 isolates) and tetracycline resistance (79 isolates).

**Conclusion:**

LA-MRSA may cause a relevant burden of disease in urban areas. Surprisingly, most infections in the present study occurred in the absence of a livestock link, suggesting inter-human transmission. These findings and the presence of PVL and other immune evasive complex virulence genes warrant future surveillance and preventative measures.

## Introduction

The past two decades have shown a rapid increase in methicillin-resistant *Staphylococcus aureus* (MRSA) strains belonging to the clonal complex 398 (CC398) in livestock, the so-called Livestock-Associated MRSA (LA-MRSA). These strains pose a zoonotic risk, particularly for those working in close contact with pigs, veal calves, and poultry (Van Den Broek et al., 2009), as colonization rates of up to 63% have been reported (Cuny et al., 2009; Van Cleef et al., 2010; Bisdorff et al., 2012; van Cleef et al., 2014). However, recent data have shown that LA-MRSA is also an emerging and significant pathogen among people outside the farming industry, and emergence of LA-MRSA was associated with an increasing number of infections of skin, soft tissue, and bloodstream (Fitzgerald, 2012; Lekkerkerk et al., 2012; Köck et al., 2013; Larsen et al., 2015, 2017).

Previous studies reported the emergence of “human” LA-MRSA sub-lineages spreading independently of a livestock reservoir (McCarthy et al., 2012; Bosch et al., 2016). Such lineages could be better adapted to the human host due to the acquisition of virulence and host adaptation genes, which are commonly located on mobile genetic elements (Matuszewska et al., 2020). Such adaptations may increase the risk of human-to-human spread in healthcare settings and in communities in non-farming areas. The European Centre for Disease Prevention and Control (ECDC) highlighted the public health and veterinary importance of regarding LA-MRSA as a ‘One Health’ issue and recommended systematic surveillance to map potential reservoirs and transmission pathways in order to enable appropriate control measures (ECDC, 2013).

While there is an urgent need to understand the various aspects of the occurrence of LA-MRSA in non-farming areas, the impact of LA-MRSA in urban areas has not yet been studied. Hence, there is a paucity of data on the clinical and infection control burden of such infections.

The objectives of this retrospective study were to assess the clinical and infection control burden in an urban area with limited livestock in Netherlands, to gain more insight into the epidemiological characteristics and the genetic diversity using Whole-Genome Sequencing (WGS), and to identify potential transmission routes of LA-MRSA in urban settings.

## Materials and methods

**Figure 1:**
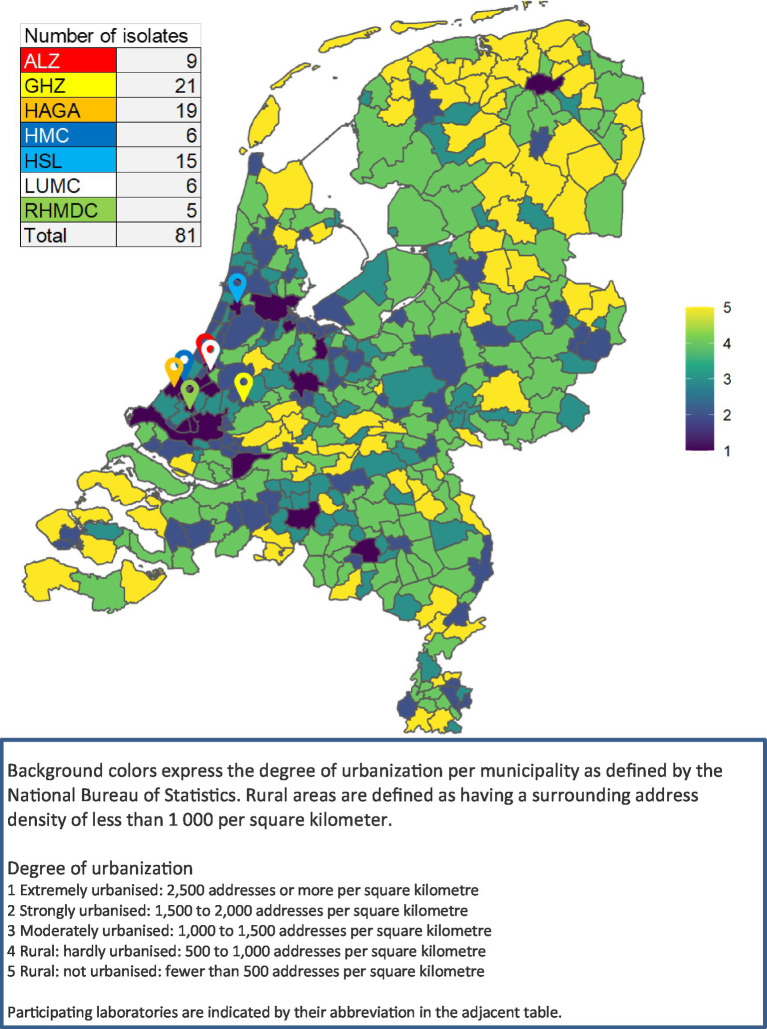
Map of Netherlands showing the urbanization level and the participating laboratories.

In this retrospective study, LA-MRSA isolates were included between 2014 and 2018 from patients who either resided in the study region or received clinical care there. The study region comprised mostly urban areas in the Mid-West region of the Netherlands (provinces Noord-Holland and Zuid-Holland). A map with the location of the laboratories and a background of rural versus urban areas as defined by the National Bureau of Statistics can be found in Figure 1 (Centraal Bureau van de Statistiek, n.d.). Seven laboratories from this study region collaborated in the study. These laboratories serve both hospitals and primary care patients.

### National surveillance

The Dutch national MRSA surveillance is a voluntary surveillance system. *Via* this system, virtually all Dutch medical microbiology laboratories (MMLs) submit their MRSA isolates for characterization and archiving to the National Institute of Public Health and the Environment (RIVM). All MRSA isolates are submitted by the MMLs *via* the Type-Ned MRSA system and are subjected to MLVA, which also includes the detection of the genes for *mecA*, *mecC*, and the *lukF* gene, which is indicative for Panton-Valentine leukocidin (PVL) (Schouls et al., 2009).

### Inclusion of LA-MRSA isolates

LA-MRSA was defined as MRSA belonging to the MLVA complex 398. Isolates submitted by six of the seven study laboratories were included through the national surveillance. One laboratory did not participate in the national surveillance, but clinical MRSA isolates in this laboratory are stored at −80°C for future reference as part of routine procedures. Hence, we used areal-time PCR to select LA-MRSA isolates for the study as described by van Meurs et al. (2013). This PCR targets the ST398-specific sequence SAPIG2194 and produces a 124-bp fragment.

### Clinical data and contact tracing

We retrospectively evaluated patient files to retrieve patient demographics, livestock exposure, and other MRSA risk factors as defined in the national guidelines. Clinical charts were reviewed to determine if an MRSA infection was present and whether cultures had been taken for screening or for clinical purposes. In case of positive clinical cultures, patient files were assessed to differentiate between infection and colonization of the sample site. Patients with exclusively positive screening cultures and/or colonization were categorized as “carrier only.” MRSA risk factors include having been an MRSA carrier in the past, having a known MRSA close contact, having received medical treatment abroad, international adoption, and livestock contact.

Baseline characteristics were compared to the data available from Type-Ned for non-LA-MRSA isolates from the participating laboratories and to nationwide data for LA-MRSA isolates. Infection control reports were assessed for results of contact tracing in the event of an unexpected MRSA finding. Data were entered into the Castor Electronic Data Capture system.

The study protocol approval and the need for patient consent were waived by the medical ethical committee of the Leiden University Medical Center (G18.021/SH/sh).

### Whole-genome sequencing and analysis

Isolates for the study were recovered from −80°C storage and grown on a blood agar plate. Several colonies totaling 50–100 μl biomass volume per isolate were suspended in 450 μl Zymo DNA/RNA Shield buffer (Zymo Research, United States) for transport to the external NGS Laboratory (BaseClear, Leiden, Netherlands). DNA extraction was done using a standard molecular biology kit from Zymo Research. Study isolates were subjected to next-generation sequencing (NGS) analysis using the Illumina HiSeq 2500 after Genomic Nextera XT library preparation NGS data were used for *de novo* assembly using CLC Genomic Workbench v20.0.3 (Qiagen Bioinformatics, Aarhus, Denmark). Isolates were characterized using a whole-genome MLST (wgMLST) scheme based on the 1,861 core-genome and 706 accessory-genome scheme as developed by SeqSphere version v3.5.0 (Ridom GmbH, Münster, Germany) with the *S. aureus COL* (NC_002951.2, 10-JUN-2013) as a reference genome. The obtained wgMLST profiles were imported into BioNumerics to assess genetic relatedness of the isolates (*7.6.3*, Applied Maths, Sint-Martens). Genetic clusters were distinguished with the SeqSphere-defined cluster alert of 24 alleles (Leopold et al., 2014). Minimum spanning trees were drawn using BioNumerics using both study isolates and NGS data available from the national surveillance (2014 to 2018) that were submitted by non-study laboratories.

Resistance and virulence genes were determined using ResFinder (version 4.0) and VirulenceFinder (*version 2.04*) databases. If the presence of certain resistance or virulence genes varied within a genetic cluster, we additionally aligned the reference genome to the WGS data of the isolate using CLC Genomics to exclude minor errors in the sequencing and assembly process.

## Results

A total of 81 patients with LA-MRSA were included in the study, which comprised 12% of all the MRSA isolates found in the study laboratories. The LA-MRSA and non-LA-MRSA isolates from the study laboratories made up 3.3% of all MRSA isolates in the national surveillance during the study period. MRSA isolates from the regional laboratories accounted for 1.6% of all nationwide LA-MRSA isolates and 3.8% of non-LA-MRSA isolates, respectively. Table 1 presents the baseline characteristics of the study patients compared to characteristics of the national LA-MRSA and regional non-LA-MRSA population.

**Table 1:** Baseline characteristics of the study patients.

Characteristic	LA-MRSA urban (*n* = 81)	LA-MRSA nationwide (*n* = 4,946)	Urban non-LA-MRSA (*n* = 640)
Age in year	0–86	0–99	0–96
-mean	*48*	*50*	*43*
Female (%)	43%	38%	44%
Carriage only (%)	68%	80%	65%
Infection/clinical sample (%)	28%	16%	33%
MRSA risk factor data available (%)	98%	51%	78%
-of whom positive^₁^	*66%*	*43%*	*41%*
-of whom had livestock contact	*26%*	*67%*	*0.2%*
-MUO^₂^	*34%*	*24%*	*47%*
PVL-positive isolates	3.7%	2.5%	37%

Urban MRSA and Nationwide LA-MRSA data derived from Type-Ned.

₁ as defined in national guidelines.

₂ MUO: MRSA of unknown origin.

Due to missing data, not all the percentages add up to 100%.

### Risk factors

Risk factors for MRSA carriage were present in 52 (66%) of the patients, 21 of whom had been in contact with livestock. Overall, 60 (76%) of the 81 included patients had no recorded livestock contact. In the 27 patients with no recorded MRSA risk factors, all LA-MRSA cultures were incidental findings that were either detected in clinical cultures (*n* = 22) or during pre-operative screenings for orthopedic joint implantation (*n* = 5). For 2 patients, there was a lack of risk factor data; however, the LA-MRSA had been cultured in an MRSA screening culture.

### Clinical presentation

Infection was present in 23 patients (28%), and an additional three patients had positive clinical cultures without apparent infection. Cultures originated mostly from the skin and soft tissue (*n* = 20), but pulmonary (*n* = 5) and urinary tract (*n* = 1) infections were also diagnosed. Five patients required some type of surgical intervention as part of the treatment. No deep-seated infections causing bacteremia occurred in the study population, while sporadic bacteremia cases (*n* = 28) did occur in the national population.

### Carriage and eradication

For 51 of the 81 included patients, follow-up cultures were available. Carriage of LA-MRSA varied from a single positive finding to carriage of up to 72 months. Eradication was successful in 38 patients and failed in 13 patients. In the group with persistent carriage (*n* = 13), the recorded carriage duration varied from 4 to 72 months. Complicating circumstances for eradication that occurred among the patients included persistent exposure in farmers and their families, chronic skin defects, or persistent use of foreign materials, such as urinary catheters. One patient in whom eradication treatment failed was in possession of a feline pet that carried an LA-MRSA strain of an identical cluster type. Eradication was only successful after simultaneous treatment of both the pet and the patient.

### Contact tracing

In 14 cases, a contact tracing investigation had been initiated by the Infection Control department. For 8 investigations, the number of screened contacts is known; these ranged from 1 to 103 contacts, with a total of 226 contacts. Nosocomial transmission was demonstrated in 2 investigations, namely Cluster 1 and 2, with eight confirmed secondary cases and two possible secondary cases (Table 2).

**Table 2:** Epidemiological background per cluster.

Genetic cluster	Epidemiological links/results of contact tracing
1	Outbreak in hospital with 9 out of 10 cases identified *via* contact tracing. The suspected index case was a nurse with atopic eczema. The positive cases detected *via* contact tracing were 6 patients and 2 of their household contacts. For one case, the route of transmission is unknown; this concerned a screening of an epidemiologically unrelated ICU patient waiting for an organ transplantation.
2	Nosocomial transmission, MRSA of index case detected in a screening prior to an orthopedic surgery and a positive contact identified *via* contact tracing. For the third patient, a dialysis patient who returned from a vacation abroad and was found to be colonized in a wound, the transmission route is unknown.
3, 5, 9	Isolates of same patient submitted by different healthcare institutions. Data are shown as a control group for the ability of cluster detection.
4, 8, 13	Epidemiological link and route of transmission unknown.
6, 10, 11, 12	Household members
7	Owner with feline pet animal. One unlinked case.

### Next-generation sequencing analysis and wgMLST

MRSA NGS data were present for 74 study patients and one pet animal. For several reasons, NGS data of seven patients were missing. Five patients provided two isolates, hence NGS data for 80 isolates were included in the study.

**Figure 2:**
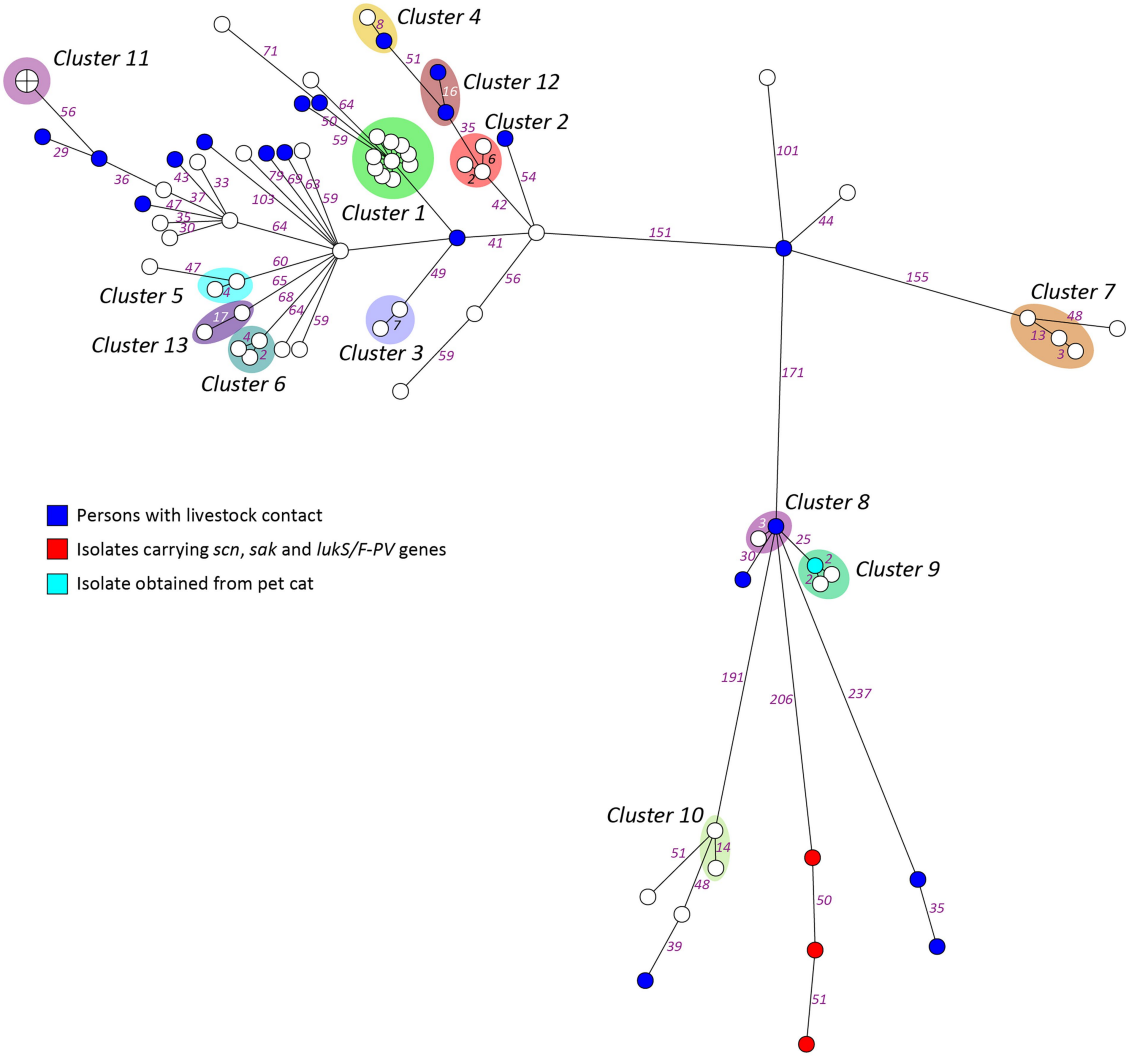
Minimum spanning tree based on wgMLST of the study isolates. Genetic distance is represented by the numbers on the connecting lines as the number of allelic differences in the wgMLST scheme.

**Figure 3:**
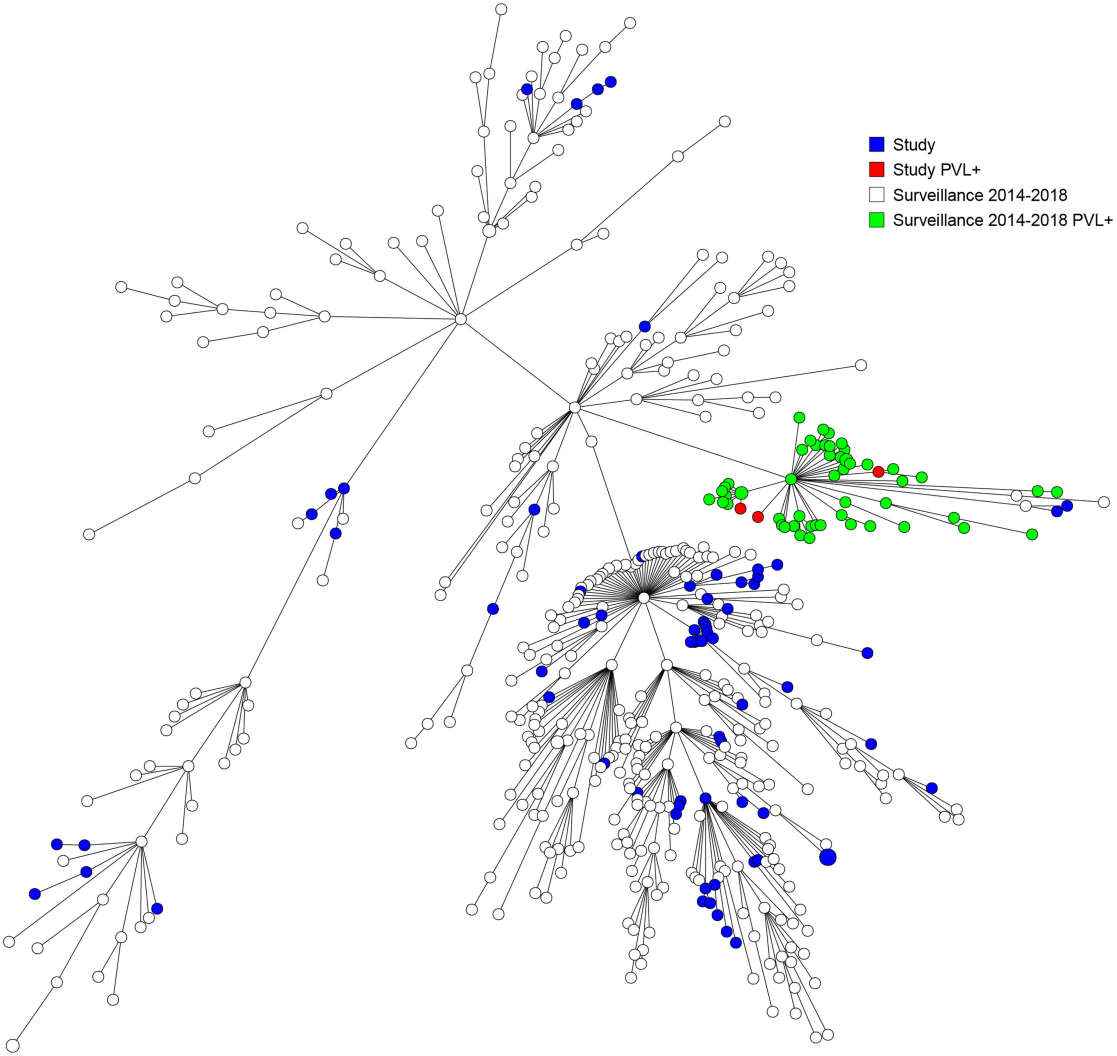
Minimum spanning tree based on wgMLST including both study and national surveillance isolates from 2014 till 2018.

A heterogenic genetic population was detected, in which many allelic differences were present (Figure 2). Analysis by means of wgMLST revealed 13 genetic clusters, which were clusters of nosocomial transmission, household members, unknown epidemiological links, and a control group of three isolates from one patient submitted by different laboratories. An overview of all the genetic clusters detected with wgMLST can be found in Table 2. An MST including nationwide LA-MRSA isolates is shown in Figure 3.

### Virulence and resistance genes

All isolates carried *hlg* and *aur* genes. Eight isolates carried additional virulence factors. Three isolates carried PVL and other genes of the immune evasive complex and were not part of a genetic cluster but showed some genetic relation within the same branch. Four isolates carried *scn*, and one isolate carried *tst*. Of these 8 patients, only one had a known livestock contact and worked in a poultry slaughterhouse. Four patients had relocated from abroad or had been abroad recently, including two patients with isolates that lacked resistance genes for tetracycline that were detected during a routine adoption screening.

All isolates carried *mecA*, and 5 isolates missed *blaZ*. Tetracycline resistance genes were detected in 78 out of 80 isolates (*tetK* 47 isolates, *tetL* seven isolates, *tetM* 74 iolates, and *tetT* one isolate). A full overview of all virulence and resistance genes detected in the study isolates is provided in the Supplementary material.

## Discussion

This study describes the molecular epidemiology of LA-MRSA in an urban region using genetic analysis based on whole-genome sequencing (WGS), a technique which has a high discriminatory power to distinguish closely related isolates in cases in which a single source or direct transmission is plausible. In our study population of 81 patients with LA-MRSA, 78% had no known livestock contact. The absence of this traditional risk factor led to a significant burden on Infection Control, with 14 contact tracing investigations due to unexpected MRSA findings, resulting in the identification of one large outbreak and a small cluster of nosocomial transmission. Three isolates carried genes coding for PVL production, and 98% of the isolates carried tetracycline resistance genes. Five genetic clusters included cases without a known epidemiological link.

Livestock-associated methicillin-resistant *Staphylococcus aureus* (LA-MRSA) carriage had a considerable impact on the patients included in our study. Almost a third of these patients had an LA-MRSA infection, and several of these infections (*n* = 5) required surgical intervention. Interestingly, the infection rate in our study population was higher than the infection rate in the national data. A limitation of the study is the missing data with regard to the presence of risk factors in the national surveillance, which makes comparison of risk factor occurrence difficult. The higher infection rate in the urban population may be due to selection bias, as patients with no risk factors will not receive screening cultures and thus remain unrecognized until an infection occurs. This would imply that the actual LA-MRSA occurrence might be even higher within the urban population than the incidence of 12% described in this study. Prolonged carriage of MRSA or failure of MRSA eradication was identified in 25% of the patients for whom data were available. More research is needed for a more complete understanding of carriage duration and eradication success rates, as this study was not designed to obtain complete follow-up data.

Whole-genome MLST identified 13 genetic clusters with allelic differences ranging from 0 to 17, and the cluster alert was set to 24. In our setting, the use of a cluster alert of up to 24 is informative. For the detection of transmission events within a short time span, such as outbreaks, other authors have suggested a threshold of up to eight allelic differences to define *S. aureus* transmission (Mellmann et al., 2016; Park et al., 2017). Indeed, most isolates in the large hospital outbreak genetic cluster in our study had 1 or 2 allelic differences, and only a single case had eight allelic differences. However, to understand the epidemiology of LA-MRSA, it is also relevant to detect transmission events over a prolonged period of time or indirect transmissions, which are likely to result in more allelic differences. We detected four genetic clusters above the threshold of eight allelic differences: three household clusters and one cluster with an unknown link. The slightly higher number of allelic differences could be attributed to the time that elapsed between the transmission event in the past and the moment of incidental discovery of carriage.

Our study population strains are a genetically diverse population that resembles the national LA-MRSA reservoir and shows no evidence of a dominant lineage. Our findings are in line with the outcomes of several studies in countries with intensive livestock farming that did not find any evidence that clonal expansion of specific sub-lineages is responsible for the increase of LA-MRSA cases among the general population. These authors suggested that the general population is consistently exposed to a random spillover of bacteria from livestock reservoirs, which are transmitted by human-to-human contact or *via* environmental transmission routes (Lekkerkerk et al., 2015; Grøntvedt et al., 2016; Larsen et al., 2017; Sieber et al., 2018, 2019). It is of interest that in our study, half of the carriers of a specific so-called “human” and more virulent CC398 strain may have acquired it abroad, as four out of eight patients had a recent link to a foreign stay. However, more study is needed to establish if there is a genetic relatedness to strains such as the ones reported from Asia (Møller et al., 2019; Lu et al., 2021).

We found several instances of human-to-human transmission. This transmission route may explain the dissemination among the urban population, although the present study did not examine the potential role of environmental transmission routes. In view of the high percentage of LA-MRSA carriage in patients without a livestock link in our study, more research is needed to elucidate the exact transmission routes.

## Conclusion

In the studied urban area in Netherlands, most of the detected LA-MRSA carriers did not have any livestock contact, while 12% of all MRSA strains were LA-MRSA as we observed human-to-human transmission, it does seem plausible that the livestock population acts as a reservoir of LA-MRSA and that continuous introduction of LA-MRSA to the human population with subsequent human-to-human transmission is the cause of urban human LA-MRSA acquisition. Contact tracings of incidental LA-MRSA findings revealed a hospital outbreak. Taking this into account, it is of concern that the present study identified PVL- and *scn/sak*-positive genetic lineages. To design potential interventions, it is important to closely monitor the occurrence of LA-MRSA and to further investigate transmission routes in urban areas. However, interventions may not be effective without eliminating the reservoir of LA-MRSA.

## Data availability statement

The WGS data presented in the study are deposited in the European Nucleotide Archive repository. The accession numbers are included in the Supplementary material Data Sheet.

## Author contributions

MMK, JG, TB, and LS: conception or design of the work. MMK, MK, SW, JaK, EM, DH, EE, JoK, VH, EC: data collection. MMK, LS, JG: data analysis and interpretation. MMK, LS: Drafting the article. Critical revision of the article and final approval of the version to be published. All authors contributed to the article and approved the submitted version.

## Funding

Funding received from NL Ministerie van Volksgezondheid, Wetenschap en Sport for the costs involved in the laboratory analysis of the study.

## Conflict of interest

The authors declare that the research was conducted in the absence of any commercial or financial relationships that could be construed as a potential conflict of interest.

## Publisher’s note

All claims expressed in this article are solely those of the authors and do not necessarily represent those of their affiliated organizations, or those of the publisher, the editors and the reviewers. Any product that may be evaluated in this article, or claim that may be made by its manufacturer, is not guaranteed or endorsed by the publisher.

## Supplementary material

The Supplementary material for this article can be found online at: https://www.frontiersin.org/articles/10.3389/fmicb.2022.875775/full#supplementary-material
