# Supplementary material for: Livestock-associated methicillin-resistant Staphylococcus aureus epidemiology, genetic diversity, and clinical characteristics in an urban region
Source: Front Microbiol. 2022 Dec 14;13:875775. doi: 10.3389/fmicb.2022.875775 (PMC9795226; doi:10.3389/fmicb.2022.875775)
Supplement: Supplementary file 1 [file Data_Sheet_1.PDF]

Livestock-Associated Methicillin-Resistant *Staphylococcus aureus* epidemiology, genetic diversity and clinical characteristics in an urban region

Supplemental Table. Overview per isolate of all detected resistance and virulance genes by ResFinder en VirulenceFinder

	aac(6')-aph	aadD	ant(6)-Ia	ant(9)-Ia	aph(2'')-Ia	aph(3')-III	str	blaZ	mecA	lnu(A)	lnu(B)	erm(47)	erm(A)	erm(B)	erm(C)	erm(T)	lnu(G)	lsa(E)	vga(A)V	fexA	vga(A)LC	vga(E)	tet(K)	tet(L)	tet(M)	tet(T)	dfrG	dfrK
RIVM_M025495	-	-	-	-	<+>	-	-	-	<+>	-	-	-	-	<+>	-	-	-	-	-	-	-	-	-	-	-	-	-	-
RIVM_M025711	<+>	-	-	-	-	-	-	<+>	<+>	-	-	-	-	-	<+>	-	-	-	-	-	-	-	-	-	<+>	-	-	<+>
RIVM_M026015	-	-	-	-	-	-	-	<+>	<+>	-	-	-	-	-	-	-	-	-	-	-	-	-	<+>	-	-	-	-	-
RIVM_M026060	-	<+>	<+>	-	-	-	-	<+>	<+>	-	<+>	-	-	-	-	-	-	<+>	-	-	-	-	<+>	-	<+>	-	<+>	-
RIVM_M026339	<+>	-	-	-	-	-	-	<+>	<+>	-	-	-	-	-	-	-	-	-	-	-	-	-	-	-	<+>	-	-	<+>
RIVM_M026364	<+>	-	-	-	-	-	-	<+>	<+>	-	-	-	-	-	<+>	-	-	-	-	-	-	-	-	-	<+>	-	-	<+>
RIVM_M026365	<+>	-	-	-	-	-	-	<+>	<+>	-	-	-	-	-	<+>	-	-	-	-	-	-	-	-	-	<+>	-	-	<+>
RIVM_M026406	<+>	-	-	-	-	-	-	<+>	<+>	-	-	-	-	-	<+>	-	-	-	-	-	-	-	-	-	<+>	-	-	<+>
RIVM_M026407	<+>	-	-	-	-	-	-	<+>	<+>	-	-	-	-	-	-	-	-	-	-	-	-	-	-	-	<+>	-	-	<+>
RIVM_M026435	<+>	-	-	-	-	-	-	<+>	<+>	-	-	-	-	-	<+>	-	-	-	-	-	-	-	-	-	<+>	-	-	<+>
RIVM_M026436	<+>	-	-	-	-	-	-	<+>	<+>	-	-	-	-	-	<+>	-	-	-	-	-	-	-	-	-	<+>	-	-	<+>
RIVM_M026453	-	-	-	-	-	-	-	<+>	<+>	-	-	-	-	-	-	-	-	-	-	-	-	-	<+>	-	<+>	-	-	-
RIVM_M026520	<+>	-	-	-	-	-	-	<+>	<+>	-	-	-	-	-	<+>	-	-	-	-	-	-	-	-	-	<+>	-	-	<+>
RIVM_M026535	<+>	-	-	-	-	-	-	<+>	<+>	-	-	-	-	-	-	-	-	-	-	-	-	-	-	-	<+>	-	-	<+>
RIVM_M026563	<+>	-	-	-	-	-	-	<+>	<+>	-	-	-	-	-	-	-	-	-	-	-	-	-	-	-	<+>	-	-	<+>
RIVM_M026601	<+>	-	-	-	-	-	-	<+>	<+>	-	-	-	-	-	<+>	-	-	-	-	-	-	-	-	-	<+>	-	-	<+>
RIVM_M026705	<+>	-	-	-	-	-	-	<+>	<+>	-	-	-	-	-	<+>	-	-	-	-	-	-	-	-	-	<+>	-	-	<+>
RIVM_M026977	-	-	-	-	-	-	-	<+>	<+>	-	-	-	-	-	-	-	-	-	-	-	-	-	<+>	-	<+>	-	-	-
RIVM_M026983	-	<+>	-	-	-	-	-	<+>	<+>	-	-	-	-	-	-	<+>	-	-	-	-	-	-	-	<+>	<+>	-	-	<+>
RIVM_M027100	-	<+>	-	-	-	-	-	<+>	<+>	-	-	-	-	-	-	<+>	-	-	-	-	-	-	-	<+>	<+>	-	-	<+>
RIVM_M027148	-	-	-	<+>	-	-	-	<+>	<+>	-	-	-	<+>	-	-	-	-	-	-	-	-	-	<+>	-	<+>	-	-	-
RIVM_M027174	-	-	-	<+>	-	-	-	<+>	<+>	-	-	-	<+>	-	-	-	-	-	-	-	-	-	<+>	-	<+>	-	-	-
RIVM_M027242	-	<+>	-	-	-	-	-	<+>	<+>	-	-	-	-	-	-	<+>	-	-	-	-	-	-	-	<+>	<+>	-	-	<+>
RIVM_M027251	-	-	-	-	-	-	-	<+>	<+>	<+>	-	-	-	-	-	-	-	-	-	-	-	-	<+>	-	<+>	-	-	-
RIVM_M027841	-	<+>	-	-	-	-	<+>	<+>	<+>	-	-	-	-	<+>	-	-	-	-	-	-	-	-	<+>	<+>	<+>	-	-	<+>
RIVM_M028270	<+>	-	-	-	-	-	-	<+>	<+>	-	-	-	-	-	-	-	-	-	-	-	-	-	-	-	<+>	-	-	<+>
RIVM_11027362	-	-	-	-	-	-	-	<+>	<+>	-	-	-	-	-	-	-	-	-	-	-	<+>	-	<+>	-	<+>	-	-	<+>
RIVM_M028947	<+>	-	-	-	-	-	-	<+>	<+>	-	-	-	-	-	-	-	-	-	-	-	-	-	-	-	<+>	-	-	<+>
RIVM_M029584	-	-	-	-	-	-	-	<+>	<+>	-	-	-	-	-	-	-	-	-	-	-	-	-	<+>	-	<+>	-	-	-
RIVM_M029727	-	-	-	-	-	-	-	<+>	<+>	-	-	-	-	-	-	-	-	-	-	-	-	-	<+>	-	<+>	-	-	-
RIVM_M029913	<+>	-	-	-	-	-	-	<+>	<+>	-	-	-	-	-	-	-	-	-	-	-	-	-	-	-	<+>	-	-	<+>
RIVM_M030552	-	-	-	-	-	-	<+>	<+>	<+>	-	-	-	-	-	<+>	-	-	-	-	-	-	-	<+>	-	<+>	-	-	-
RIVM_M030954	-	-	-	-	-	-	-	<+>	<+>	-	-	-	-	-	-	-	<+>	-	-	-	-	-	<+>	-	<+>	-	<+>	-
RIVM_M031204	-	-	-	-	-	-	-	<+>	<+>	-	-	-	-	-	-	-	-	-	-	-	-	-	<+>	-	<+>	-	<+>	-
RIVM_M031283	-	-	-	-	-	-	-	<+>	<+>	-	-	-	-	-	-	-	-	-	-	-	-	-	<+>	-	<+>	-	-	-
RIVM_M031540	-	-	-	-	-	-	-	-	<+>	-	-	-	-	-	-	-	-	-	<+>	-	-	-	-	-	<+>	-	-	-
RIVM_11030094	-	-	-	-	-	-	-	-	<+>	-	-	-	-	-	-	-	-	-	<+>	-	-	-	-	-	<+>	-	-	-
RIVM_M031656	<+>	-	-	-	-	-	<+>	<+>	<+>	-	-	-	-	-	-	-	-	-	-	-	-	-	-	-	<+>	-	-	<+>
RIVM_M031826	-	-	-	-	-	-	-	<+>	<+>	-	-	-	-	-	-	-	-	-	-	-	-	-	<+>	-	<+>	-	-	-
RIVM_M031862	-	-	-	-	-	-	-	<+>	<+>	-	-	-	-	-	-	-	-	-	-	-	-	-	<+>	-	<+>	-	-	-
RIVM_M032007	-	-	-	-	-	-	-	<+>	<+>	-	-	-	-	-	-	-	-	-	-	-	-	-	<+>	-	<+>	-	-	-
RIVM_M032032	-	-	-	-	-	-	-	-	<+>	-	-	-	-	-	-	-	-	-	-	-	-	-	-	-	-	-	-	-
RIVM_M032034	-	-	-	-	-	-	-	<+>	<+>	-	-	-	-	-	-	-	-	-	-	-	-	-	<+>	-	<+>	-	-	-
RIVM_M032066	-	-	-	-	-	-	-	<+>	<+>	-	-	-	-	-	<+>	-	-	-	-	<+>	-	-	<+>	-	<+>	-	<+>	-
RIVM_M032336	-	-	-	-	-	-	-	<+>	<+>	-	-	-	-	-	-	-	-	-	-	-	-	-	<+>	-	<+>	-	-	<+>
RIVM_M032481	-	-	-	-	-	-	-	<+>	<+>	-	-	-	-	-	-	-	-	-	-	-	-	-	<+>	-	<+>	-	-	<+>
RIVM_M032482	-	-	-	-	-	-	-	<+>	<+>	-	-	-	-	-	-	-	-	-	-	-	-	-	<+>	-	<+>	-	-	<+>
RIVM_M032484	-	-	-	-	-	-	<+>	<+>	<+>	-	-	-	-	-	<+>	-	-	-	-	-	-	-	<+>	-	<+>	-	-	-
RIVM_M033174	-	-	-	<+>	-	-	-	<+>	<+>	-	-	-	<+>	-	-	-	-	-	-	-	-	<+>	<+>	-	<+>	-	<+>	-
RIVM_M033244	-	-	-	-	-	-	-	<+>	<+>	-	-	-	-	-	-	-	-	-	-	-	-	-	<+>	-	<+>	-	-	-
RIVM_M033354	-	-	-	-	-	-	-	<+>	<+>	-	-	-	-	-	-	-	-	-	-	-	-	-	<+>	-	<+>	-	<+>	-
RIVM_M033553	<+>	-	-	-	-	-	-	<+>	<+>	-	-	-	-	-	-	-	-	-	-	-	-	-	-	-	<+>	-	-	<+>
RIVM_M033590	-	<+>	-	-	-	-	-	<+>	<+>	-	-	-	-	-	-	-	-	-	-	-	-	-	<+>	<+>	<+>	-	-	<+>
RIVM_M033644	-	-	-	-	-	-	-	<+>	<+>	-	-	-	-	-	-	-	<+>	-	-	-	-	-	<+>	-	<+>	-	-	-
RIVM_M033881	<+>	-	-	-	-	-	-	<+>	<+>	-	-	-	-	-	<+>	-	-	-	-	-	-	-	-	-	<+>	-	-	<+>
RIVM_M033941	<+>	-	-	-	-	-	-	<+>	<+>	-	-	-	-	-	-	-	-	-	-	-	-	-	-	-	<+>	-	-	<+>
RIVM_M034064	<+>	-	-	-	-	-	-	<+>	<+>	-	-	-	-	-	-	-	-	-	-	-	-	-	-	-	<+>	-	-	<+>
RIVM_M034180	-	-	-	<+>	-	-	-	<+>	<+>	-	-	-	<+>	-	-	-	-	-	-	-	-	<+>	<+>	-	<+>	-	<+>	-
RIVM_M034527	-	-	-	-	-	-	-	<+>	<+>	-	-	-	-	-	-	-	-	-	-	<+>	-	-	<+>	-	<+>	-	-	-
RIVM_M034650	-	-	-	-	-	-	-	<+>	<+>	-	-	-	-	-	-	-	-	-	-	-	-	-	<+>	-	<+>	-	-	-
RIVM_M034722	-	-	-	-	-	-	-	<+>	<+>	-	-	-	-	-	-	-	-	-	-	-	-	-	<+>	-	<+>	-	-	<+>
RIVM_M035131	<+>	-	-	-	-	-	-	<+>	<+>	-	-	-	-	-	-	-	-	-	-	-	-	-	-	-	<+>	-	-	<+>
RIVM_M035215	-	-	-	-	-	-	-	<+>	<+>	-	-	-	-	-	-	-	<+>	-	-	-	-	-	<+>	-	<+>	-	<+>	-
RIVM_M035512	-	-	-	<+>	-	-	-	<+>	<+>	-	-	-	<+>	-	-	-	-	-	-	-	-	-	<+>	-	-	-	-	-
RIVM_M036335	<+>	-	-	-	-	-	-	<+>	<+>	-	-	-	-	-	<+>	-	-	-	-	-	-	-	-	-	<+>	-	-	<+>
RIVM_M036829	-	-	-	<+>	-	-	-	<+>	<+>	-	-	-	<+>	-	-	-	-	-	-	-	-	-	<+>	-	-	-	-	-
RIVM_M037578	-	-	-	-	-	-	-	<+>	<+>	-	-	-	-	-	-	-	<+>	-	-	-	-	-	<+>	-	<+>	-	<+>	-
RIVM_M037664	-	-	-	<+>	-	-	<+>	<+>	<+>	-	-	-	<+>	-	-	-	-	-	-	-	-	<+>	-	-	<+>	-	<+>	-
RIVM_11036592	-	-	-	-	-	-	<+>	<+>	<+>	-	-	-	-	-	-	-	<+>	-	-	-	-	-	<+>	-	<+>	-	<+>	-
RIVM_M038425	<+>	-	-	-	-	-	-	<+>	<+>	-	-	-	-	-	-	-	-	-	-	-	-	-	-	-	<+>	-	-	<+>
RIVM_M038590	-	-	<+>	-	-	<+>	-	<+>	<+>	-	-	<+>	-	-	<+>	-	-	-	-	-	-	-	<+>	-	<+>	<+>	-	<+>
RIVM_M039149	-	-	-	<+>	-	-	-	<+>	<+>	-	-	-	<+>	-	-	-	-	-	-	-	-	-	<+>	-	<+>	-	-	-
RIVM_M039353	-	-	-	<+>	-	-	-	<+>	<+>	-	-	-	<+>	-	-	-	-	-	-	-	-	-	<+>	-	-	-	-	-
RIVM_M039740	-	-	<+>	-	-	-	-	<+>	<+>	-	<+>	-	-	-	-	-	-	<+>	-	-	-	-	<+>	-	<+>	-	-	-
RIVM_M039884	-	-	<+>	-	-	-	-	<+>	<+>	-	<+>	-	-	-	-	-	-	<+>	-	-	-	-	<+>	-	<+>	-	-	-
RIVM_M039898	<+>	<+>	-	-	-	-	-	<+>	<+>	-	-	-	-	-	<+>	<+>	-	-	-	-	-	-	-	<+>	<+>	-	-	<+>
RIVM_M044677	-	<+>	-	-	-	-	-	<+>	<+>	-	-	-	-	-	-	-	-	-	-	-	-	-	<+>	<+>	<+>	-	-	<+>
17000285	-	-	-	-	-	-	<+>	<+>	<+>	-	-	-	-	-	-	-	<+>	-	-	-	-	-	<+>	-	<+>	-	<+>	-
17000287	-	-	-	-	-	-	-	<+>	<+>	-	-	-	-	-	-	-	-	-	-	-	-	-	<+>	-	<+>	-	-	-
17000288	-	-	-	-	-	-	-	-	<+>	-	-	-	-	-	-	-	-	-	-	-	-	-	<+>	-	<+>	-	-	-

		sea	sec	sec3	sek	sel	seq	aur	tst	hlgA	hlgB	hlgC	sak	scn	lukF-PV	lukS-PV
RIVM_M025495	11023969	-	<+>	<+>	-	<+>	-	<+>	-	<+>	<+>	<+>	<+>	<+>	-	-
RIVM_M025711	11024185	-	-	-	-	-	-	<+>	-	<+>	<+>	<+>	<+>	<+>	-	-
RIVM_M026015	11024520	-	-	-	-	-	-	<+>	-	<+>	<+>	<+>	-	-	-	-
RIVM_M026060	11024565	-	-	-	-	-	-	<+>	-	<+>	<+>	<+>	-	-	-	-
RIVM_M026339	11024844	-	-	-	-	-	-	<+>	-	<+>	<+>	<+>	-	-	-	-
RIVM_M026364	11024869	-	-	-	-	-	-	<+>	-	<+>	<+>	<+>	-	-	-	-
RIVM_M026365	11024870	-	-	-	-	-	-	<+>	-	<+>	<+>	<+>	-	-	-	-
RIVM_M026406	11024911	-	-	-	-	-	-	<+>	-	<+>	<+>	<+>	-	-	-	-
RIVM_M026407	11024912	-	-	-	-	-	-	<+>	-	<+>	<+>	<+>	-	-	-	-
RIVM_M026435	11024940	-	-	-	-	-	-	<+>	-	<+>	<+>	<+>	-	-	-	-
RIVM_M026436	11024941	-	-	-	-	-	-	<+>	-	<+>	<+>	<+>	-	-	-	-
RIVM_M026453	11024958	-	-	-	-	-	-	<+>	-	<+>	<+>	<+>	-	-	-	-
RIVM_M026520	11025025	-	-	-	-	-	-	<+>	-	<+>	<+>	<+>	-	-	-	-
RIVM_M026535	11025040	-	-	-	-	-	-	<+>	-	<+>	<+>	<+>	-	-	-	-
RIVM_M026563	11025068	-	-	-	-	-	-	<+>	-	<+>	<+>	<+>	-	-	-	-
RIVM_M026601	11025106	-	-	-	-	-	-	<+>	-	<+>	<+>	<+>	-	-	-	-
RIVM_M026705	11025210	-	-	-	-	-	-	<+>	-	<+>	<+>	<+>	-	-	-	-
RIVM_M026977	11025482	-	-	-	-	-	-	<+>	-	<+>	<+>	<+>	-	-	-	-
RIVM_M026983	11025488	-	-	-	-	-	-	<+>	-	<+>	<+>	<+>	-	-	-	-
RIVM_M027100	11025605	-	-	-	-	-	-	<+>	-	<+>	<+>	<+>	-	-	-	-
RIVM_M027148	11025653	-	-	-	-	-	-	<+>	-	<+>	<+>	<+>	-	-	-	-
RIVM_M027174	11025679	-	-	-	-	-	-	<+>	-	<+>	<+>	<+>	-	-	-	-
RIVM_M027242	11025747	-	-	-	-	-	-	<+>	-	<+>	<+>	<+>	-	-	-	-
RIVM_M027251	11025756	-	-	-	-	-	-	<+>	-	<+>	<+>	<+>	-	-	-	-
RIVM_M027841	11026347	-	-	-	-	-	-	<+>	-	<+>	<+>	<+>	-	-	-	-
RIVM_M028270	11026776	-	-	-	-	-	-	<+>	-	<+>	<+>	<+>	-	-	-	-
RIVM_11027362	11027362	-	-	-	-	-	-	<+>	-	<+>	<+>	<+>	-	-	-	-
RIVM_M028947	11027453	-	-	-	-	-	-	<+>	-	<+>	<+>	<+>	-	-	-	-
RIVM_M029584	11028090	-	-	-	-	-	-	<+>	-	<+>	<+>	<+>	-	-	-	-
RIVM_M029727	11028233	-	-	-	-	-	-	<+>	-	<+>	<+>	<+>	-	-	-	-
RIVM_M029913	11028419	-	-	-	-	-	-	<+>	-	<+>	<+>	<+>	-	-	-	-
RIVM_M030552	11029058	-	-	-	-	-	-	<+>	-	<+>	<+>	<+>	-	-	-	-
RIVM_M030954	11029460	-	-	-	-	-	-	<+>	-	<+>	<+>	<+>	-	-	-	-
RIVM_M031204	11029710	-	-	-	-	-	-	<+>	-	<+>	<+>	<+>	-	-	-	-
RIVM_M031283	11029789	-	-	-	-	-	-	<+>	-	<+>	<+>	<+>	-	-	-	-
RIVM_M031540	11030046	-	-	-	-	-	-	<+>	-	<+>	<+>	<+>	-	-	-	-
RIVM_11030094	11030094	-	-	-	-	-	-	<+>	-	<+>	<+>	<+>	-	-	-	-
RIVM_M031656	11030162	-	-	-	-	-	-	<+>	-	<+>	<+>	<+>	-	-	-	-
RIVM_M031826	11030332	-	-	-	-	-	-	<+>	-	<+>	<+>	<+>	-	-	-	-
RIVM_M031862	11030368	-	-	-	-	-	-	<+>	-	<+>	<+>	<+>	-	-	-	-
RIVM_M032007	11030513	-	-	-	-	-	-	<+>	-	<+>	<+>	<+>	-	-	-	-
RIVM_M032032	11030538	-	<+>	<+>	-	<+>	-	<+>	-	<+>	<+>	<+>	<+>	<+>	-	-
RIVM_M032034	11030540	-	-	-	-	-	-	<+>	-	<+>	<+>	<+>	-	-	-	-
RIVM_M032066	11030572	-	-	-	-	-	-	<+>	-	<+>	<+>	<+>	-	-	-	-
RIVM_M032336	11030842	-	-	-	-	-	-	<+>	-	<+>	<+>	<+>	-	-	-	-
RIVM_M032481	11030987	-	-	-	-	-	-	<+>	-	<+>	<+>	<+>	-	-	-	-
RIVM_M032482	11030988	-	-	-	-	-	-	<+>	-	<+>	<+>	<+>	-	-	-	-
RIVM_M032484	11030990	-	-	-	-	-	-	<+>	-	<+>	<+>	<+>	-	-	-	-
RIVM_M033174	11031680	-	-	-	-	-	-	<+>	<+>	<+>	<+>	<+>	-	-	-	-
RIVM_M033244	11031750	-	-	-	-	-	-	<+>	-	<+>	<+>	<+>	-	-	-	-
RIVM_M033354	11031860	-	-	-	-	-	-	<+>	-	<+>	<+>	<+>	-	-	-	-
RIVM_M033553	11032059	-	-	-	-	-	-	<+>	-	<+>	<+>	<+>	-	-	-	-
RIVM_M033590	11032096	-	-	-	-	-	-	<+>	-	<+>	<+>	<+>	-	-	-	-
RIVM_M033644	11032150	-	-	-	-	-	-	<+>	-	<+>	<+>	<+>	-	-	-	-
RIVM_M033881	11032387	-	-	-	-	-	-	<+>	-	<+>	<+>	<+>	-	-	-	-
RIVM_M033941	11032447	-	-	-	-	-	-	<+>	-	<+>	<+>	<+>	-	-	-	-
RIVM_M034064	11032570	-	-	-	-	-	-	<+>	-	<+>	<+>	<+>	-	-	-	-
RIVM_M034180	11032686	-	-	-	-	-	-	<+>	-	<+>	<+>	<+>	-	-	-	-
RIVM_M034527	11033033	-	-	-	-	-	-	<+>	-	<+>	<+>	<+>	-	-	-	-
RIVM_M034650	11033156	-	-	-	-	-	-	<+>	-	<+>	<+>	<+>	-	-	-	-
RIVM_M034722	11033228	-	-	-	-	-	-	<+>	-	<+>	<+>	<+>	-	-	-	-
RIVM_M035131	11033637	-	-	-	-	-	-	<+>	-	<+>	<+>	<+>	-	-	-	-
RIVM_M035215	11033721	-	-	-	-	-	-	<+>	-	<+>	<+>	<+>	-	-	-	-
RIVM_M035512	11034018	-	-	-	-	-	-	<+>	-	<+>	<+>	<+>	<+>	<+>	<+>	<+>
RIVM_M036335	11034843	<+>	-	-	<+>	-	<+>	<+>	-	<+>	<+>	<+>	-	<+>	-	-
RIVM_M036829	11035340	-	-	-	-	-	-	<+>	-	<+>	<+>	<+>	<+>	<+>	<+>	<+>
RIVM_M037578	11036089	-	-	-	-	-	-	<+>	-	<+>	<+>	<+>	-	-	-	-
RIVM_M037664	11036175	-	-	-	-	-	-	<+>	-	<+>	<+>	<+>	-	-	-	-
RIVM_11036592	11036592	-	-	-	-	-	-	<+>	-	<+>	<+>	<+>	-	-	-	-
RIVM_M038425	11036936	-	-	-	-	-	-	<+>	-	<+>	<+>	<+>	-	-	-	-
RIVM_M038590	11037101	-	-	-	-	-	-	<+>	-	<+>	<+>	<+>	-	-	-	-
RIVM_M039149	11037660	-	-	-	-	-	-	<+>	-	<+>	<+>	<+>	-	-	-	-
RIVM_M039353	11037864	-	-	-	-	-	-	<+>	-	<+>	<+>	<+>	<+>	<+>	<+>	<+>
RIVM_M039740	11038251	-	-	-	-	-	-	<+>	-	<+>	<+>	<+>	-	-	-	-
RIVM_M039884	11038395	-	-	-	-	-	-	<+>	-	<+>	<+>	<+>	-	-	-	-
RIVM_M039898	11038409	-	-	-	-	-	-	<+>	-	<+>	<+>	<+>	-	-	-	-
RIVM_M044677	11043212	-	-	-	-	-	-	<+>	-	<+>	<+>	<+>	-	-	-	-
17000285	17000285	-	-	-	-	-	-	<+>	-	<+>	<+>	<+>	-	-	-	-
17000287	17000287	-	-	-	-	-	-	<+>	-	<+>	<+>	<+>	-	-	-	-
17000288	17000288	-	-	-	-	-	-	<+>	-	<+>	<+>	<+>	-	-	-	-
